# Cardiac magnetic resonance T1 and extracellular volume mapping with motion correction and co-registration based on fast elastic image registration

**DOI:** 10.1007/s10334-017-0668-2

**Published:** 2017-12-21

**Authors:** Shuo Zhang, Thu Thao Le, Sven Kabus, Boyang Su, Derek J. Hausenloy, Stuart A. Cook, Calvin W. L. Chin, Ru San Tan

**Affiliations:** 1 National Heart Centre Singapore, 5 Hospital Drive, Singapore, 169609 Singapore; 2 Philips Healthcare Singapore, 622 Lorong 1, Singapore, 319763 Singapore; 3 Philips Research, Röntgenstrasse 24-26, 22335 Hamburg, Germany; 4 Duke-NUS Medical School, 8 College Road, Singapore, 169857 Singapore; 5 The Hatter Cardiovascular Institute, University College London, London, UK; 6 The National Institute of Health Research University College London Hospitals Biomedical Research Centre, London, UK

**Keywords:** T1 relaxation time, Extracellular volume, Motion correction, Co-registration, Diffuse fibrosis

## Abstract

**Objective:**

Our aim was to investigate the technical feasibility of a novel motion compensation method for cardiac magntic resonance (MR) T1 and extracellular volume fraction (ECV) mapping.

**Materials and methods:**

Native and post-contrast T1 maps were obtained using modified look-locker inversion recovery (MOLLI) pulse sequences with acquisition scheme defined in seconds. A nonrigid, nonparametric, fast elastic registration method was applied to generate motion-corrected T1 maps and subsequently ECV maps. Qualitative rating was performed based on T1 fitting-error maps and overlay images. Local deformation vector fields were produced for quantitative assessment. Intra- and inter-observer reproducibility were compared with and without motion compensation.

**Results:**

Eighty-two T1 and 39 ECV maps were obtained in 21 patients with diverse myocardial diseases. Approximately 60% demonstrated clear quality improvement after motion correction for T1 mapping, particularly for the poor-rating cases (23% before vs 2% after). Approximately 67% showed further improvement with co-registration in ECV mapping. Although T1 and ECV values were not clinically significantly different before and after motion compensation, there was improved intra- and inter-observer reproducibility after motion compensation.

**Conclusions:**

Automated motion correction and co-registration improved the qualitative assessment and reproducibility of cardiac MR T1 and ECV measurements, allowing for more reliable ECV mapping.

**Electronic supplementary material:**

The online version of this article (10.1007/s10334-017-0668-2) contains supplementary material, which is available to authorized users.

## Introduction

Myocardial fibrosis is one of the histological hallmarks of left-ventricular decompensation [1]. Although myocardial biopsy is the gold standard for diagnosing fibrosis, the procedure is invasive and susceptible to sampling error. Instead, late gadolinium-enhanced imaging, a conventional cardiovascular magnetic resonance (CMR) technique, offers accurate detection of focal regions of myocardial fibrosis. However, this form of fibrosis commonly occurs late in the disease process and is not believed to be reversible [2, 3]. Recent advances in myocardial T1 mapping allow quantification of more diffuse types of fibrosis, which are potentially reversible with targeted therapies [4]. Conversely, unlike native (non-contrast) myocardial T1 that reflects disease involving both the cardiomyocyte and interstitium, extracellular volume fraction (ECV) directly estimates interstitial expansion [5]. In the absence of amyloid deposition and edema, ECV correlates with the amount of myocardial fibrosis on histology [6, 7]. Moreover, recent data have also demonstrated an important prognostic association between ECV and adverse outcomes, independent of traditional predictors such as ejection fraction, age, and coronary artery disease [4, 8].

State-of-the-art CMR for quantitative T1 mapping relies on electrocardiogram (ECG)-triggered acquisitions following initial magnetization preparation, e.g., inversion recovery (IR). One of the most widely used methods is the modified look-locker inversion recovery (MOLLI) technique [9]. However, all clinically available techniques, including MOLLI, result in a series of images across multiple heart beats within one breath hold, which is susceptible to motion artifacts caused by inconsistent breath-holding and varying cardiac cycles. Furthermore, current ECV estimation relies on cumbersome measurement of myocardial and blood-pool T1 before and after gadolinium contrast administration, rendering it prone to image misalignment due to two separate acquisitions. Therefore, an automated process of motion correction and co-registration of native and post-contrast images, as well as maps, is critical for developing T1 and ECV mapping for clinical applications. Despite previous works [10, 11], such an approach remains unavailable universally in clinical practice on all vendor platforms. The purpose of this work was to investigate the technical feasibility and clinical performance of a novel straightforward and robust approach for motion compensation at 3T.

## Materials and methods

### Patients

Twenty-one patients [18 men, 3 women, age range 17–71 (mean 47 ± 18) years] with diverse cardiac pathologies underwent CMR on a 3T system (Ingenia, Philips Healthcare, Best, The Netherlands) with a maximum 28-channel body (12-channel spine, 16-channel torso) coil. Standard CMR protocol included multislice, multidirectional survey images, steady-state free precession (SSFP) cine gradient-echo sequences at two-, three-, and four-chamber views, and short-axis slices covering the left ventricle from base to apex. For all sequences, coil-element combination was automatically determined for efficient signal-to-noise ratio (SNR) in the selected field of view (FOV), with measured coil sensitivity in the reference scan at the beginning of the examination for each patient [12]. All patients gave written informed consent before each magnetic resonance imaging (MRI) examination.

### MOLLI MRI

All T1 acquisitions were based on the modified MOLLI pulse sequence with balanced steady-state free-precession (SSFP) readout [9]. The recently proposed 5s(3s)3s and 4s(1s)3s(1s)2s schemes were applied for pre- and 20-min post-contrast (Gadovist 0.1 mmol/kg) T1 measurements, respectively [13]. The native MOLLI protocol consisted of two IR-prepared ECG-gated acquisitions (typically 8–14 images in total) separated by an interval lasting a minimum of 3 s to ensure full recovery of longitudinal magnetization, all performed within one breath-hold. Similarly, the post-contrast MOLLI protocol consisted of three IR-prepared acquisitions with two intervals of at least one second interspersed in between. Images were acquired at the basal and mid-cavity short-axis levels of the left ventricle, and all (both pre- and post-contrast) were timed to the mid-diastolic phase of the cardiac cycle and performed at end expiration. Imaging parameters were: (FOV 340 × 340 mm^2^, matrix size 188 × 168, acquired pixel size 1.8 × 2.0 mm^2^, interpolated pixel size 1.3 × 1.3 mm^2^, slice thickness 8 mm, repetition time (TR) 2.3 ms, echo time (TE) 1.15 ms, flip angle (FA) 30°, minimal interval time TI delay 87.7 ms, bandwidth 1136.5 Hz/pixel, partial Fourier factor 0.8, sensitivity encoding (SENSE) factor 2.2, scan time ~11- to 13 s [14]. Simplified pulse sequence diagrams are shown in Supplementary Fig. 1.

**Fig. 1:**
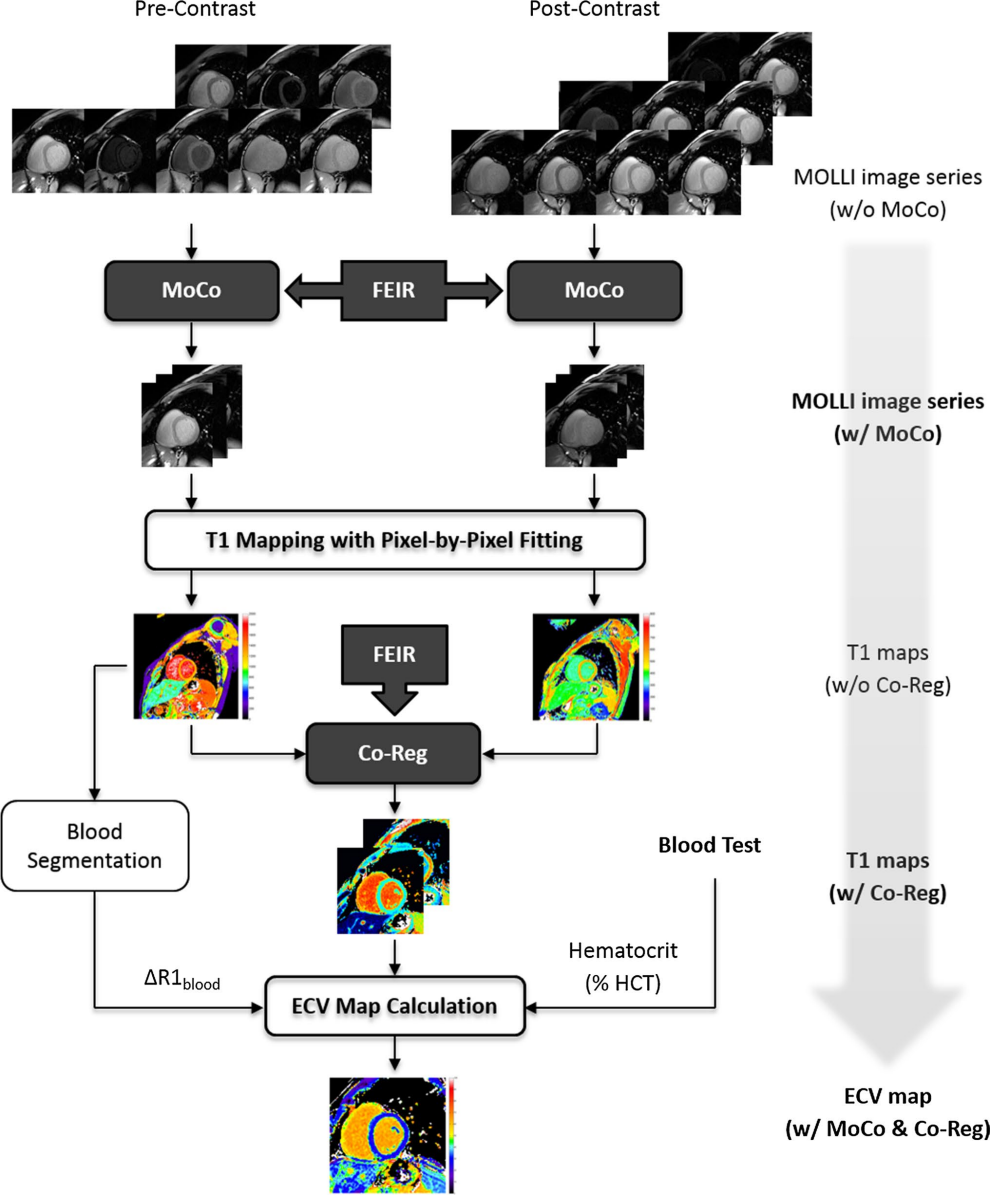
Workflow of T1 and extracellular volume (ECV) mapping combined with motion correction and co-registration using the proposed approach.* MoCo* motion correction,* Co-Reg* co-registration. For details, see text

### Motion correction and co-registration

A modified nonrigid, nonparametric image registration method was applied to generate motion-corrected MOLLI image series and subsequently to co-register the native and post-contrast T1 maps [15]. In principle, motion estimation between two images is known as an image registration or co-registration task. Given two images, a registration algorithm aims to find a displacement vector field such that its application to the first image results in a new deformed image being most similar to the second image. While this procedure is directly applicable to the alignment of native and post-contrast T1 maps, it can also be used to generate the motion-corrected MOLLI image series such that one designated image out of the series is defined as a reference frame, to which all remaining images are aligned subsequently. For motion correction of a MOLLI CMR image series, the last time frame with the longest inversion time was used as the reference image. For co-registration of native and post-contrast T1 maps, the former was used as reference image. The mathematical framework is detailed as follows:

Given two images $R,T$ defined on an image domain $\varOmega$  (here a subset of ${\mathbb{R}}^{2}$), a registration algorithm aims to find a displacement field $u: \varOmega \to {\mathbb{R}}^{2}$ such that $T\left( {id + u} \right)$ is most similar to $R$. In mathematical terms, the similarity is described by a function ${\mathcal{D}}\left[ {u,R,T} \right]$, here chosen as the normalized gradient field (NGF),

1 $${\mathcal{D}}\left[ {u,R,{\text{T}}} \right] = \frac{1}{2}\mathop \int \limits_{\varOmega }^{{}} 1 - \langle n\left( {R,x} \right),n\left( {T,x + u} \right)\rangle^{2} {\text{d}}x,$$

where the term $n(I,x)$ describes the normalized image gradient of an image $I$ at position $x$, and is given by $n(I,x):= \frac{\nabla I\left( x \right)}{\parallel{\nabla I\left( x \right)\parallel_{\varepsilon } }}$ with $\parallel x\parallel_{\varepsilon } := \left( {x_{1}^{2} + x_{2}^{2} + \varepsilon^{2} } \right)^{1/2}$. $\varepsilon$ is introduced not only to avoid a division by zero but also to control what can be interpreted as a significant difference to separate image noise from valuable information. It is chosen in relation to the total mass of edges in the image by computing the spatial gradient of the input image, taking its modulus and integrating over the entire image domain.

A registration based only on a similarity measure may yield a deformed template image that perfectly matches the reference image as long as all intensity values are present in both images. However, the problem is ill-posed, and the underlying deformation in general may not be applicable in a physical context. Therefore, an additional smoothness constraint (or regularizer) is considered, which can be chosen to model application-specific physical properties. It should be noted that the regularization term also serves as a check for the resulting vector field against unwanted physiologically implausible behaviors (e.g., strong local expansion, compression, or even folding). In addition, with regularization, the registration algorithm is less affected by image noise, imaging artifacts or abrupt change of contrast (e.g., caused by IR). In our experiments, we employed a regularizer based on the popular linear elastic potential

2 $${\mathcal{S}}\left[ u \right] = \mathop \smallint \limits_{\varOmega }^{{}} \left( {\frac{\mu }{4}\mathop \sum \limits_{k,l = 1}^{2} \left( {\partial_{{x_{k} }} u_{l} \left( x \right) + \partial_{{x_{l} }} u_{k} \left( x \right)} \right)^{2} + \frac{\lambda }{2}\left( {\nabla \cdot u\left( x \right)} \right)^{2} } \right){\text{d}}x.$$

Its elastic properties are modeled by Lamé parameters $\lambda$, $\mu$, which can be translated into Young’s modulus (linked to tissue stiffness) and Poisson’s ratio (contraction perpendicular to applied stretch). Specifically, $\mu$ is inversely proportional to the elastic modulus and $\lambda /\mu$ is proportional to the incompressibility of the material. To address respiratory and cardiac motions during the scan procedure, on the one hand, and the inflow of contrast agent on the other hand, a relatively large Young’s modulus and small Poisson’s ratio were chosen. As is common with registration algorithms, absolute values from the literature cannot be used, since their choice depends on the intensity scale of the acquired scans, the chosen similarity measure, and implementation issues.

By combining the similarity measure and the regularizing term, the problem can be formulated as finding a displacement field  $u$, which minimizes the joint function

3 $${\mathcal{T}}\left[ {u,R,T} \right] = {\mathcal{D}}\left[ {u,R,T} \right] + {\mathcal{S}}\left[ u \right].$$

The computation of the Gâteaux derivative of Eqs. (1) and (2) yields a necessary condition for $u$ being a minimizer of Eq. (3). The outcome is a system of nonlinear partial differential equations equipped with associated boundary conditions. For its discretization, finite differences in conjunction with Neumann boundary conditions were chosen. The resulting system of linear equations consists of, on the one hand, a sparse, symmetric, and highly structured matrix arising from the regularizer and, on the other hand, a so-called force vector corresponding to the similarity measure. The system of equations is then linearized and iteratively solved by a conjugate gradient scheme. The iteration is stopped if the update in $u$ is below some threshold for all positions indicating convergence. To ensure convergence and to increase robustness, the algorithm was embedded into a multiresolution scheme. This means that for each input frame, a pyramid of images is created consisting of the image at original resolution and its down-sampled copies at coarser resolutions. The registration starts at the coarsest resolution level. As soon as convergence is reached, the optimized displacement field is up-sampled to the next finer-resolution level and used as the starting point for registration on this level. This process is repeated until the finest resolution level is reached. Such a multiresolution scheme not only speeds up the entire registration process, since most computations are done on smaller images, it also increases robustness and convergence speed, with the major structures aligned first, followed by the more granular structures.

### Myocardial T1 and ECV mapping

By obtaining the motion-corrected MOLLI series, the T1 map was generated via the pixel-wise curve fitting using the three-parameter signal model [17] (Eq. 4).

4 $$S\left( t \right) = A - B \cdot e^{{ - \frac{t}{{T1^{*} }}}} .$$

*S*(*t*) described the measured T1 signal, while the unknown parameters (A, B, T1*) at each pixel was fitted using the Levenberg–Marquardt minimization [18] algorithm. The obtained apparent T1, denoted as T1*, was then corrected for signal saturation according to [17]

5 $$T1 = \left( {\frac{B}{A} - 1} \right) \cdot T1^{*} .$$

Contouring the epi- and endomyocardial borders was done manually. Further blood-pool segmentation was achieved based on native T1 map using a threshold of 1400 ms, as T1 of the pre-contrast blood ranges between 1600 and 2200 ms at 3T [3]. The ECV map was generated based on co-registered native and post-contrast T1 maps and individual hematocrit measured on the same day of the CMR examination [7, 19, 20]. For comparison, an additional ECV map was generated for each case based on motion-corrected T1 maps but without co-registration. Median native and post-contrast blood T1 values were used in the calculation of ECV according to:

6 $$ECV = \frac{{\Delta R1_{\text{myocardium}} }}{{\Delta R1_{\text{blood pool}} }} \cdot \left( { 1- {\text{hematocrit}}} \right).$$

where

7 $$\Delta R1 = \frac{1}{{T1_{\text{post}} }} - \frac{1}{{T1_{\text{pre}} }}.$$

The whole procedure followed the recently published consensus statement [5]. In addition, a T1-fitting error map with a unit of milliseconds was produced based on the estimated standard deviation (SD) of the corresponding T1-map value at each pixel, as proposed by [16]. Meanwhile, an overlay map was generated from a pair of native and post-contrast T1 maps for quality evaluation [14]. All calculation was done offline using a Matlab program developed in-house (The Mathworks, Natick, MA, USA). The entire process is shown in Fig. 1 as a straightforward approach.

### Evaluation

Performance of motion correction and image co-registration was evaluated both qualitatively by visual inspection and quantitatively. First, two observers (1 cardiologist and 1 cardiac imaging scientist with 10 and 5 years of experience, respectively) assessed the quality of T1 (native and post-contrast) and ECV maps based on the generated error and overlay maps, respectively. A 3-point ordinal score (1 = good quality, 2 = fair quality and 3 = poor quality) was used for all cases, independent from motion compensation. Subsequently, by comparing scores, the performance of the motion correction and co-registration was categorized as either improved, maintained, or deteriorated.

In addition, to evaluate the co-registration performance of native and post-contrast T1 maps for ECV mapping, the local deformation vector field $\varphi (x) = x + u(x)$, $x \in \varOmega$ was generated as an output from the registration algorithm. It established a pixel-wise correspondence between the two input images; i.e., it described for every pixel $x$ in the one image a vector pointing to the corresponding anatomical position $x + u(x)$ in the other image. Based on that, a qualitative local deformation field (LDF) map and a quantitative local volume change (LVC) map were calculated. The LDF map was calculated as ${\text{LDF(}}x )\text{ := }C(\varphi (x))$, $x \in \varOmega$, with $C:\varOmega \to {\mathbb{R}}^{2}$ being a checkerboard image, which was used to visualize local deformation. Meanwhile, as LVC was mathematically defined as the determinant of the Jacobian of the deformation vector field, for each pixel $x$ of a 2D image, it was computed as ${\text{LVC(}}x )\text{ := }{\text{det(}}\nabla \varphi (x)) - 1$, with the spatial derivatives of $\varphi$ evaluated in a small environment, consisting of 3 × 3 pixels in this work, to quantify the local volume change, including compression and expansion. A value of 0 indicated volume preservation, while a positive value was defined as expansion and a negative value as compression, not considering the through-plane motion. Both LDF and LVC maps were overlaid with the isocontour of the native T1 map as reference for visual guidance. An example underlining the principle was given in Supplementary Fig. 2 using numeric simulation.

Myocardial T1 and ECV values were measured by placing a region of interest (ROI) conservatively within the short-axis left-ventricular myocardium at the mid-myocardial region to avoid contamination by the blood pool. In addition, focal lesions with positive late gadolinium enhancement (LGE) were excluded from the ROI.

### Statistics

Data were presented as percentages for categorical variables and mean ± SD for continuous variables. The distribution of all continuous variables was tested for normality using the Shapiro–Wilk test. Intra- and inter-observer reproducibility before and after motion correction and co-registration were assessed using Bland–Altman analysis and intraclass correlation coefficients (ICC). All statistical analyses were performed using IBM SPSS Statistics version 17. A *P* < 0.05 was considered statistically significant.

## Results

We analyzed 82 myocardial T1 maps (pre- and post-contrast basal and mid-cavity slices) from 21 patients with diverse cardiac pathologies: cardiomyopathies [dilated cardiomyopathy (DCM), *n* = 6; hypertrophic cardiomyopathy (HCM), *n* = 3; restrictive cardiomyopathy, *n* = 4), ischemic heart disease (IHD) with infarct (*n* = 6), and hypertensive heart disease (*n* = 2)].

### T1 mapping with motion correction

**Fig. 2:**
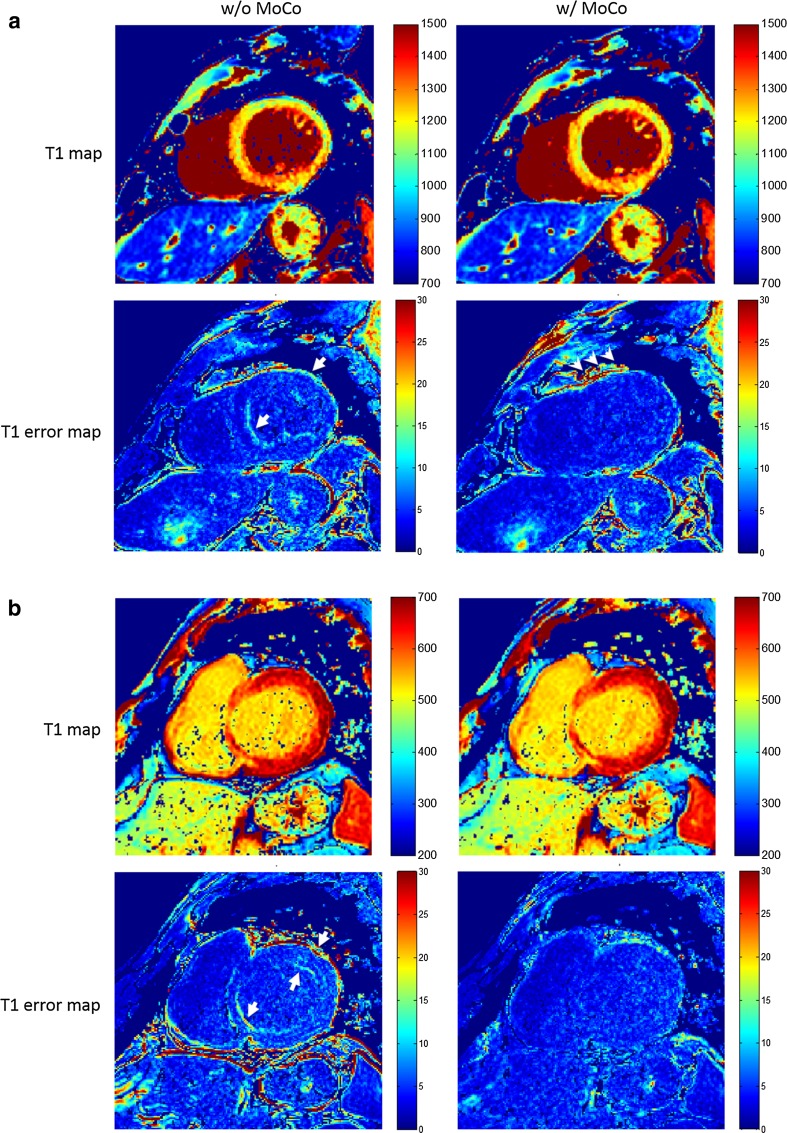
Motion correction for T1 mapping with quality improvement. Short-axis mid native (**a**) and basal post-contrast (**b**) ventricular T1 (top row) and fitting error (bottom row) maps of one patient with dilated cardiomyopathy (DCM) before (left column) and after (right column) motion correction. Myocardial structure showed better alignment compared with obvious fitting error at the septal (**a**) and septal-to-anterior (**b**) subendocardial segments without motion correction (white arrow)

**Fig. 3:**
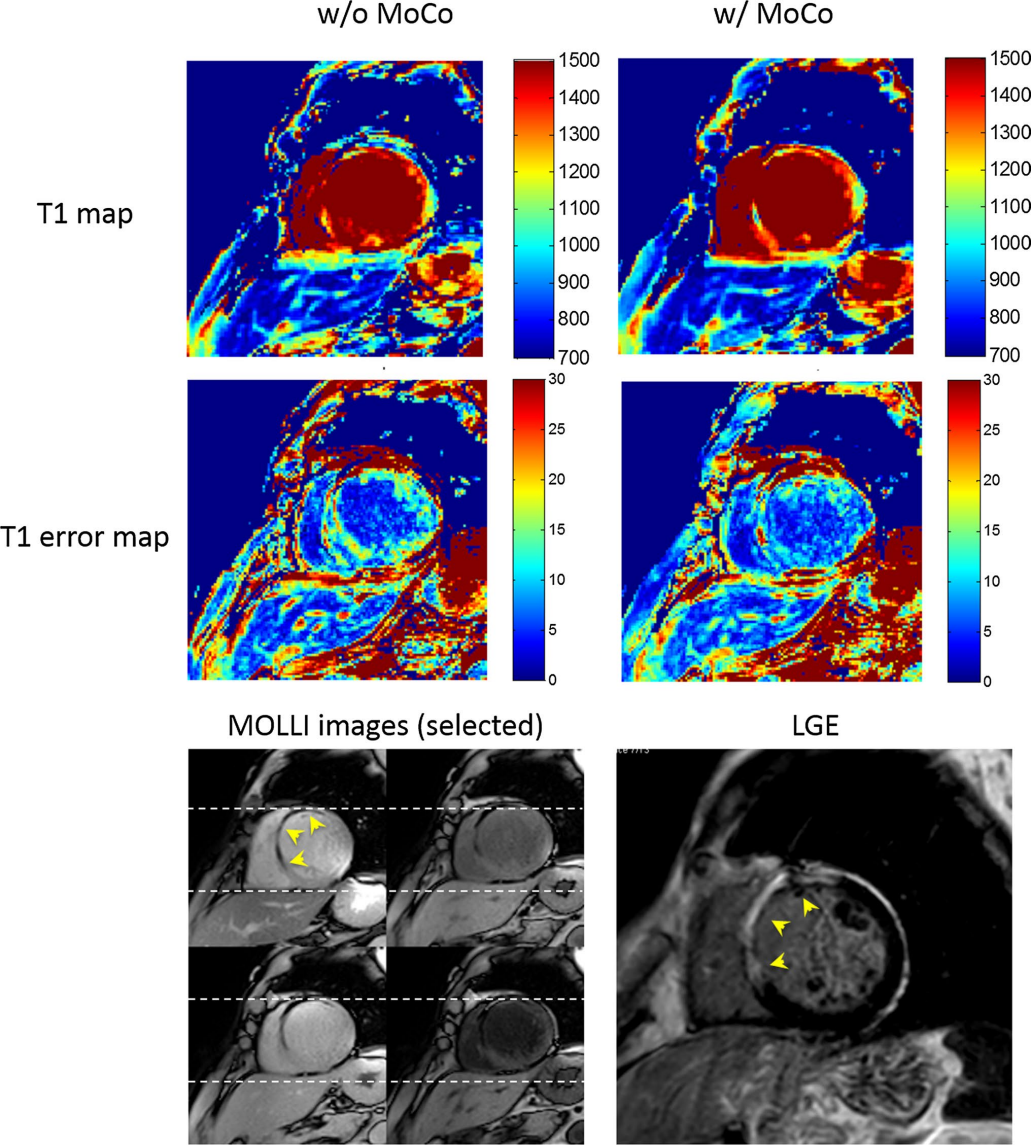
Motion correction for T1 mapping without quality improvement (from poor to poor). Short-axis mid ventricular native T1 (top row) and fitting-error (middle row) maps of one patient with ischemic heart disease (IHD) before (left column) and after (right column) motion correction. Selected modified look-locker inversion recovery (MOLLI) images (4 out of 8) showed both great motion (dotted lines) and extensive infarct, which was consistent with the late gadolinium enhancement (LGE) image (arrowheads). Two out of 82 cases remained of poor quality with motion correction and were inadequate for contouring and were thus excluded for quantitative analysis. See text for details

Table 1 summarizes the performance of motion correction on T1 mapping. Motion correction improved the quality in 59% of all maps, with a trend toward greater improvement in the post-contrast case compared with the native case (*P* = 0.07). Overall, there was an increase in good cases, from ~10% (8/82) to 48% (39/82), and a decrease of poor cases, from 23% (19/82) to 2% (2/82). Only two native cases exhibiting poor quality did not show improvement and were removed from further processing and quantitative analysis. No maps deteriorated in quality after motion correction. Selected clinical examples are presented in Figs. 2 and 3 of patients with DCM and IHD. In general, myocardial structure and shape were more aligned in the motion-corrected T1 maps with less signal variations across the myocardium. Meanwhile, there were less-visible fitting errors, as shown in error maps. A separate example with manual myocardium segmentation in video format can be found in the Supplementary Movie 1.

**Table 1:** Evaluation of motion correction (MoCo) performance for T1 mapping

	Native (*n* = 41)			Post-contrast (*n* = 41)			Total (*n* = 82)		
	Poor	Fair	Good	Poor	Fair	Good	Poor	Fair	Good
w/o MoCo	9	27	5	10	28	3	19	55	8
w/ MoCo	2	21	18	0	20	21	2	41	39
Improvement	78%	–		100%	–		89%	–	
	49% (20/41)			68% (28/41)			59% (48/82)

Myocardial T1 values of 1280 ± 51 ms native and 597 ± 73 ms post-contrast were obtained after motion correction. Reduced T1 fitting errors were found across all patients after motion correction (Supplementary Table 1).

### ECV mapping with co-registration

**Fig. 4:**
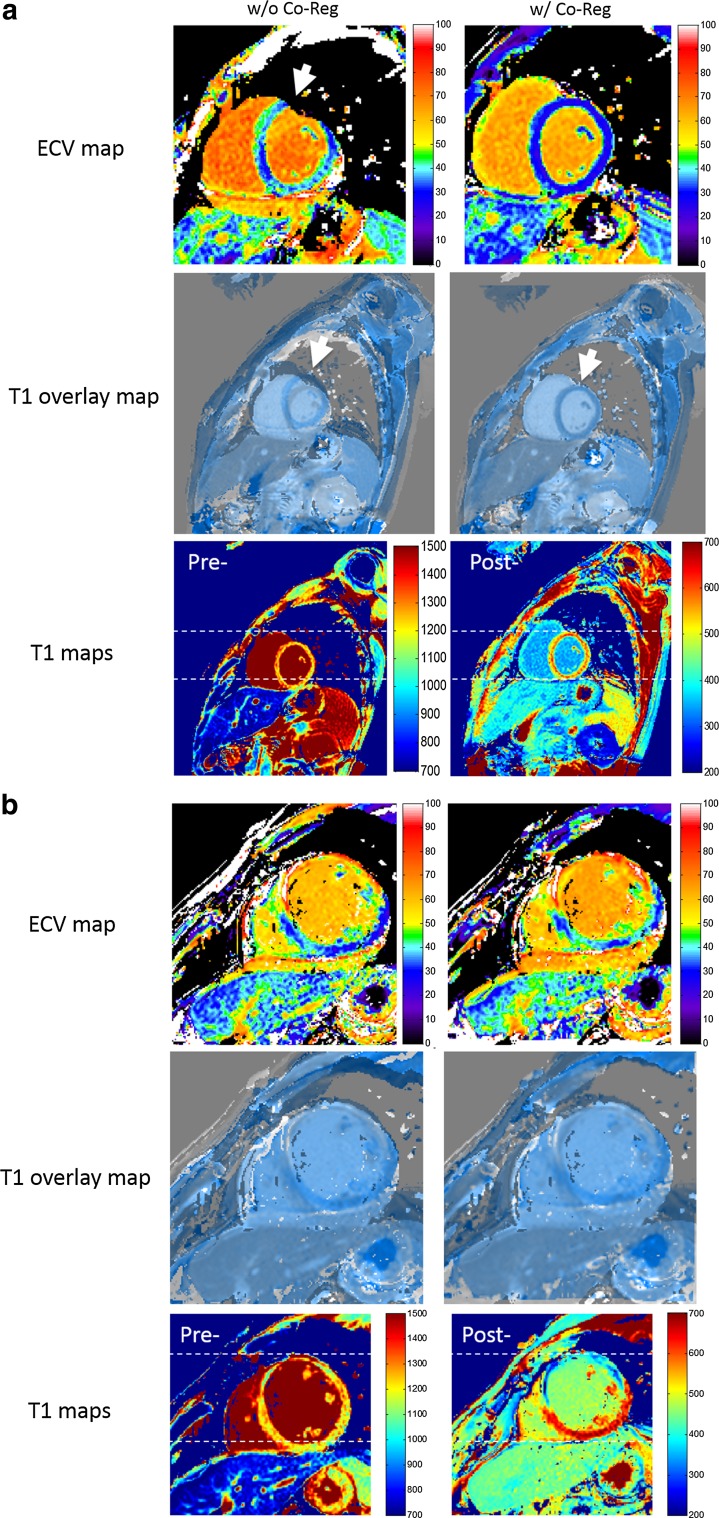
Image co-registration for extracellular volume (ECV) mapping with (**a**) and without (**b**) quality improvement. Short-axis basal ventricular ECV (top row) and native and post-contrast T1 overlay (second row) maps before (left column) and after (right column) image co-registration (Co-Reg) in two patients (**a**, **b**) with ischemic heart disease. The individual native and post-contrast T1 maps are shown side-by-side (last row), respectively. In (a), obvious misalignment seen in the T1 overlap map (white arrows) and individual native and post-contrast maps (dotted lines) was corrected with co-registration that yielded a more homogenous ECV map. In (**b**), image co-registration did not improve interpreter-assessed quality of the ECV map. Distinct motion status (dotted lines) with myocardial infarct involving the anterior and anteroseptal walls was appreciated in greater relief in the post-contrast compared with native T1 map (last row)

Based on motion-corrected native and post-contrast cardiac T1 maps, co-registration further improved the quality of ECV maps in 67% of the 39 cases and 85% of poor cases (Table 2). Ten ECV maps improved from poor to good, whereas three remained poor after co-registration. Two corresponding examples are shown in Fig. 4. Similar to motion correction, myocardial structure and shape appeared more intact in the co-registered ECV map, with less intensity variations across the myocardium.

**Table 2:** Evaluation of co-registration (Co-Reg) performance for ECV mapping

	Poor	Fair	Good
w/o Co-Reg (*n* = 39)	20	15	4
w/ Co-Reg (*n* = 39)	3	13	23
Improvement	85%	–	
	67% (26/39)

**Fig. 5:**
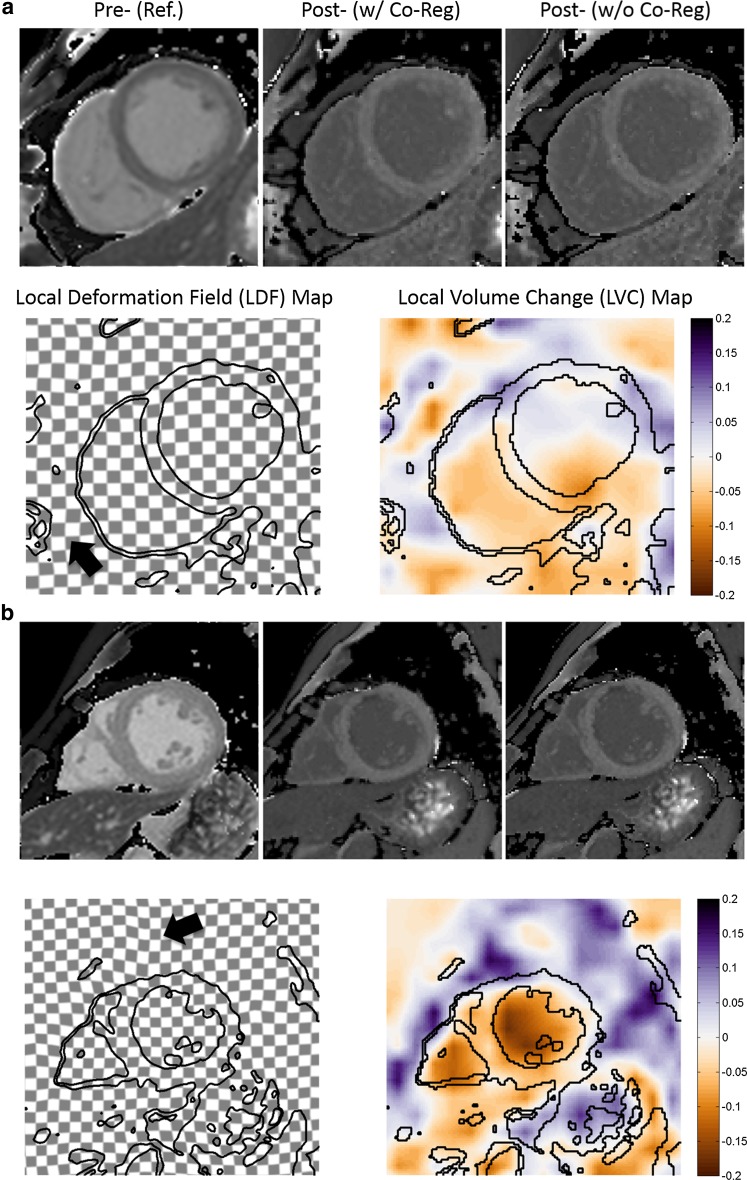
Image co-registration for extracellular volume (ECV) mapping with quality maintained. Short-axis mid ventricular T1 maps (top row), local deformation field (LDF) map (bottom left), and volume change map (bottom right) in two patients (**a**, **b**) with ischemic heart disease. For co-registration, the native T1 map (top left) served as reference and the post-contrast T1 map (top right) as target; the final map is displayed (top middle). In both cases, only small and smooth deformation were found in both the LDF checkerboard by slightly deviated vertical or horizontal lines (arrows) and local volume change (LVC) map (≤ 20%). See text for details

In regard to evaluation of the co-registration performance, generally only mild and smooth local deformation was observed, as indicated by the slightly deformed checkerboard grids in the LDF maps (Fig. 5). Meanwhile, only small local volume change (≤ 10% for myocardium) was found for most of cases, indicating good volume preservation.

In these patients with cardiac pathologies, global ECV with co-registration was 34.8 ± 7.0%, slightly higher than without co-registration (33.5 ± 7.6%). For details, see Supplementary Table 1.

### Reproducibility

**Fig. 6:**
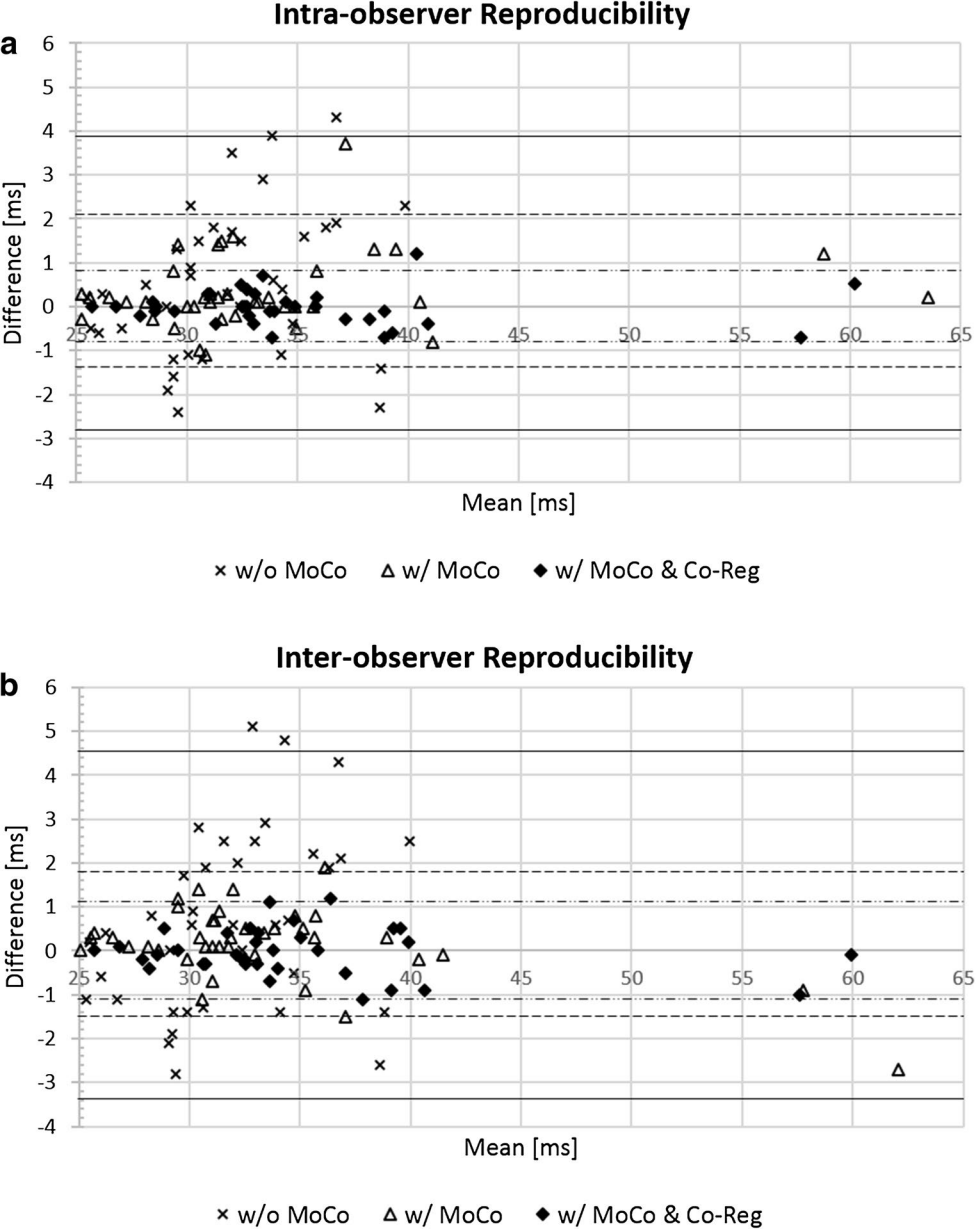
Intra- (**a**) and inter-observer (**b**) reproducibility of extracellular volume fraction (ECV) measurements without and with motion correction and co-registration. Bland–Altman plots of measured ECV values at the mid- and basal levels (*n* = 82 cases), with 95% limits of agreement (LOA) superimposed on the chart for without motion correction or co-registration (w/o MoCo, solid lines), with motion correction only (w/MoCo, dashed line), and with both motion correction and co-registration (w/MoCo and CoReg, dash–dotted line). Improved agreement was found using the proposed method, as shown by the decreased differences and tighter 95% LOA

In general, the proposed motion correction and co-registration approach resulted in improved intra- and inter-observer agreement, as presented in Fig. 6. A detailed summary showing decreased differences and higher intraclass correlation coefficients (ICC) can be found in Supplementary Table 2.

## Discussion

A method of motion correction and co-registration for myocardial T1 and ECV mapping was proposed and validated using patient data. The novel algorithm based on a fully automatic, nonrigid, nonparametric, image registration approach provides a robust solution, with improved precision and reproducibility and simplified workflow despite the widely varying contrast across the multiple inversion-recovery source images.

We show that this approach leads to improved image quality in 67% of all cases and 85% of poor cases and reduces errors without image quality deterioration. The average values of T1 and ECV before and after motion correction and co-registration differed only by a few milliseconds or percent points that, although statistically significant, may be deemed clinically insignificant (Supplementary Table 1). Of note, we demonstrate improved intra-observer reproducibility and inter-observer agreement by using the proposed method. This has important implications for reliable measurement for delineating the ranges in health and disease, as well as longitudinal surveillance of disease progression or treatment response. Additionally, increased precision translates into the need for fewer sample numbers to demonstrate statistical differences in research studies [24].

A different approach reported previously computes a synthetic MOLLI series based on estimated T1 IR signal for intra-series motion correction and further generates individually corrected post-contrast images based on a composite motion deformation field for inter-series co-registration in an iterative manner [10, 11]. Its advantage was demonstrated by comparison with a direct, nonrigid co-registration method using local cross correlation for image-similarity measure and regularization of the velocity of the displacement, which—while offering more flexibility in terms of deformation—may lead to undesired distortion. In contrast, the proposed method here applies normalized gradient field (NGF) for similarity measure and the Navier-Lamé operator as a regularizer. While the former tries to align edge orientation regardless of the direction and extent of intensity changes (described below), the latter was strongly weighted to restrict elasticity and avoid large local deformation. In addition, the proposed method applies a straightforward and stepwise process to produce motion-corrected T1 and ECV maps without iterative computation or synthetic image estimation (Fig. 1). This greatly simplifies workflow and computational demands and avoids error propagation that may occur at any (pre-) processing step. Because the correction method is independent of the outcome measure (in this case, T1 estimation), the technique can be easily extended to other MOLLI schemes, signal readouts, and even free-breathing applications [22]. In terms of computational demand, for co-registration of two 2D images (e.g., with a matrix size of 352 × 352 pixels), this method requires 0.13 s, while for alignment of 8–14 images, it typically requires 0.9–1.7 s.

The optimized MOLLI sequences with the recently proposed second-based schemes were used in this work to measure pre- and post-contrast myocardial T1 relaxation times. In comparison with the original schemes defined in beats, scan time depends less on heart rate and allows better sampling of the relaxation curve at high heart rate [13, 16]. While a thorough investigation of this scheme is beyond the scope of this study, which focused on motion correction and co-registration, two examples of pulse sequence diagrams with different heart rates are shown in Supplementary Fig. 1 for native and post-contrast T1 mapping, respectively. In addition, a moderate FA of 30° was used for reduced off-resonance sensitivity with sufficient SNR on 3T [13, 21].

The task of aligning two images during motion correction or co-registration processes requires a definition of similarity in mathematical terms. For images in which the intensity describes a quantity, such as absorption in computed tomography (CT) data, this approach is valid. For other imaging modalities, such as MRI, positron emission tomography/single-photon emission tomography (PET/SPECT), or ultrasound (US) imaging, intensities will vary. Mutual information is often described as a similarity measure suited for multimodal registration applications. When studying the way a human observer inspects and compares images, attention focuses on image structures rather than intensities. Such structures are described by image edges. Two images appear similar to each other if edges occur at the same spatial positions, and edges are similar to each other if their spatial image gradients are similar. In this work, the similarity measure was done by using NGF [23]. Although NGF relies on edge structures, its advantage here is that the orientations of the gradient vectors do not have to be the same in order to allow intensity gradients from bright to dark to match with those from dark to bright. In addition, the amplitudes of the gradient vectors do not need to be similar, so that less-contrasted edges can match with more distinctive edges. This is a necessity of the MOLLI image series, where image intensities and contrasts can change drastically during IR. Therefore, unlike previous work in motion compensation for myocardial perfusion, where multiple computed images were used as templates or reference frames due to their gradual contrast uptake in different tissue compartments [25, 26], only one image—typically the last time frame—from the MOLLI series was used as the reference in this work.

A similar image registration approach was previously applied in CT and PET imaging for 3D data, where the sum of squared differences (SSD) were used as similarity measure [15]. In our work, this was replaced by NGF, and the framework was modified and extended for co-registration of multiple 2D images, and is applied in quantitative cardiac MR parametric mapping. While there was a nearly five-fold increase in good cases and 12-fold decrease in poor cases for motion correction of the MOLLI series (Table 1), 67% of cases demonstrated further qualitative improvement for ECV mapping with co-registration of native and post-contrast T1 maps. In both scenarios, no quality deterioration was observed. Qualitative image assessment suggests better myocardial alignment with fewer errors at the endocardial and epicardial borders with motion correction compared to without (arrows in Fig. 2). More fitting errors were visible outside the heart after motion correction in this example (arrowheads in Fig. 2) but were not seen in other cases. These observations may need to be investigated in a larger cohort. Robustness and accuracy can be further demonstrated in LDF and LVC maps. In nonrigid registration, a small and smooth local deformation is often desired to avoid unwanted distortions and possible image deterioration. The computed local deformation field was used in this work to generate two independent maps for assessing the co-registration performance both qualitatively and quantitatively. In general, only mild and smooth local deformations were found in all cases (as shown by the LDF maps in Fig. 5). In particular, we did not observe a local mixed pattern of compression and expansion. A global translation neither deforms vertical or horizontal lines nor changes the size or shape of any checkerboard grids. In contrast, a local deformation may result in deformed lines and even, in case of volume change, expanded or compressed checkerboard grids. In addition, LVC was close to 0 (≤ 10%) in the myocardium, which indicated good volume preservation. Relatively larger changes (≥ 10% but ≤ 50%) were sometimes observed in either the blood pool or the lung, which might indicate different respiratory statuses and/or cardiac phases between two T1 maps (LVC maps in Fig. 5).

Two cases of motion correction of MOLLI series and three of co-registration for ECV mapping showed no improvement and remained of poor quality (Figs. 3 and 4). In these cases, both greater in- and through-plane motions and focal infarcts were observed. While such focal pathologies limited in spatial extent may not be captured in the 2D frame due to through-plane motion [27], they may lead to substantially differently recognized structural shapes that violate the physiologically plausible behavior the regularizing term is based on in the elastic modeling, thus constituting the main cause of a failed measure for similarity. It is worth mentioning that in this case, local deformation maps may not fully address the challenge, as other than misalignment of focal lesions, both in- and through-plane motions, will appear as overall mild volume changes. However, co-registration is, by design, mainly capable of correcting for in-plane motion and does not address new features or tissue structures introduced by through-plane motion.

The combination of motion correction and co-registration not only allows for more robust alignment of the myocardial structures but also improves precision for T1 calculation, as shown by the reduced T1 fitting errors (Supplementary Table 1) and better intra- and inter-observer reproducibility (Fig. 6 and Supplementary Table 2). In addition, it helps improve the workflow for ECV mapping. A pixel-by-pixel calculation of the ECV map provides direct visualization and may facilitate easy interpretation for clinical studies. Further technical improvement will focus on parameter optimization, computational power, synthetic ECV mapping without hematocrit [28, 29], and, in particular, an integrated process. A straightforward and stepwise procedure of the proposed method also facilitates easy integration of the algorithm on the MR scanner, in addition to its robustness against large variations of image contrast throughout IR.

This initial report may pave the way for a more comprehensive evaluation of the proposed method on a larger clinical cohort. Moreover, the proposed method may be extended to other parametric mapping and to free-breathing image series, such as perfusion or cine data, which need to be investigated in future studies.

This work has several limitations. First, this was an exploratory study on the technical feasibility of the proposed method, and only a small number of patients was included. Nevertheless, we demonstrate enhanced reproducibility of T1 and ECV measurements by motion correction and co-registration, respectively. Second, diverse cardiac pathologies were studied. Relatively higher T1 and ECV values were found in the cohort in comparison with those previously reported [30, 31] but cannot be taken to be representative of individual pathologies or considered as normal ranges, as values are predictably affected by different flip angles and other variations in vender-specific implementation. Due to scan-time restriction, only the aforementioned second-based MOLLI scheme was used in this study. A thorough investigation of it in comparison with other existing methods may be warranted in a larger clinical cohort. Moreover, in common with all motion correction and co-registration techniques, blood signal contamination in the myocardium is a source of potential error. In the current work, this error was minimized by manual ROI definition, including only the mid-myocardial region for analysis, leaving a wide margin at the epicardial and endocardial borders. In addition, image smoothing and through-plane motion remain challenging problems for all image registration methods, and further studies are needed. Focal macrofibrosis evident on LGE images that are likely to be present in some patients may result in heterogeneous regional T1 measurements within the slice, which was not specifically addressed in this study. A local reference based on the same sequence settings needs to be established to further investigate its clinical significance; however, it was not the focus of this study. Finally, the presence of irregular heartbeats may degrade image quality, which has neither been investigated in this study nor in works by others on MOLLI-based sequences.

## Conclusion

This work describes a robust motion correction and co-registration method for both myocardial T1 and ECV mapping based on a fully automatic, nonrigid, nonparametric, image registration approach. Improved image quality, precision, and reproducibility have been demonstrated. The simple workflow allows for seamless integration into the image acquisition and reconstruction pipeline for future clinical practice. Further research is needed to validate aspects of both novel MOLLI variants and the proposed motion compensation approach in a larger cohort.

## Electronic supplementary material

Below is the link to the electronic supplementary material.

Supplementary material 1 (DOCX 13 kb)

Supplementary material 2 (PNG 115 kb) Supplementary Fig. 1 Schematic diagram of the modified look-locker inversion recovery (MOLLI) pulse sequence for myocardial T1 mapping. For native T1 mapping with a 5s(3s)3s scheme and enhanced or post-contrast mapping with a 4s(1s)3s(1s)2s scheme [13], different heartrates of 50 and 120 bpm were illustrated, respectively. The schemes including the look-locker (LL) periods and recovery intervals are essentially defined in terms of time periods with minimum durations in seconds. For instance, the number of single-shot image acquisitions during the LL periods is the ratio of defined minimum time duration to the R–R interval in seconds, rounded up to the next higher integer (e.g., 5 and 3 in** A**; 8, 6, and 4 in** B**), while recovery time is the minimum period between the end of the last acquired image to the next electrocardiogram (ECG)-triggered inversion recovery (IR) that encompasses a delay of ≥3 s for native (A) and of ≥1 s for postcontrast (B). Unlike the conventional beat-based scheme, the number of acquired images during each LL varies according to heartrate: less when R–R interval is longer; more when shorter. The exact scan time for breath-hold varies only slightly with the actual heartrate (13.2 s for 50 bpm using the native scheme and 10 s for 120 bpm using the enhanced scheme, as shown here), and is typically ~10 to 13 s.* IR* inversion recovery pulse, * LL* look-locker,* RO* signal readout

Supplementary material 3 (TIFF 534 kb) Supplementary Fig. 2 Example of local deformation during image co-registration based on numeric simulation. A reference image (*top left*) was created with two circles of equal size. The image (*top right*) was created with larger and smaller diameters for the corresponding circles, respectively, and was registered to the reference image. The warped image after co-registration (*top middle*) showed compression and expansion from the corresponding circles, respectively. This can also be visualized by the locally deviated horizontal and vertical lines in the local deformation field (LDF) checkerboard map, as well as by the values in the local volume change (LVC) map, where a* negative sign* and* brown color coding* indicates compression (getting “hotter”), while a* positive sign* and* blue color coding* indicates expansion (getting “colder”). Such compression and expansion are purely image-based terms, which may be required for changes in the cross-sectional tissue area caused by motion. Both maps were overlaid with the iso-contours of the reference image. The same approach was applied to data and figures in this work

Supplementary material 4 (AVI 3460 kb) Supplementary Movie 1: Motion correction for T1 mapping with quality improvement. Short-axis mid-ventricular native modified look-locker inversion recovery (MOLLI) series of one patient with dilated cardiomyopathy (DCM) without (*left*) and with (*right*) motion correction. Myocardium segmentation was done manually from the first image and taken as a mask. Better tissue alignment along the MOLLI series can be seen after motion correction

## Abbreviations

CMR: Cardiovascular magnetic resonance
bSSFP: Balanced steady-state free precession
ECG: Electrocardiogram
ECV: Extracellular volume fraction
LGE: Late gadolinium enhancement
LV: Left ventricular
MOLLI: Modified look-locker inversion recovery
T1: Longitudinal relaxation time constant
