# Supplementary material for: Cardiac magnetic resonance T1 and extracellular volume mapping with motion correction and co-registration based on fast elastic image registration
Source: MAGMA. 2017 Dec 21;31(1):115–29. doi: 10.1007/s10334-017-0668-2 (PMC5813095; doi:10.1007/s10334-017-0668-2)
Supplement: Supplementary file 1 — Supplementary material 1 (DOCX 13 kb) [file 10334_2017_668_MOESM1_ESM.docx]

**Supplementary Table 1: Myocardial T1 values, fitting errors, and ECV of patients before and after motion compensation on 3T (n = 41).**

|  | Native T1  [ms] | Post-contrast T1  [ms] | T1 fitting error  [ms] | ECV  [%] |
| --- | --- | --- | --- | --- |
| w/o MoCo or  Co-Reg | 1282.5 ± 62.1* | 617.5 ± 63.5 | 7.1 ± 1.6* | 33.5 ± 7.6 |
| w/ MoCo or  Co-Reg | 1280.4 ± 51.1 | 596.7 ± 62.9 | 3.7 ± 0.9 | 34.8 ± 7.0 |
| P | < 0.005^#^ | < 0.001 | < 0.001^#^ | < 0.05 |

* Unlike the rest of the groups, native T1 and T1 fitting error had non-normal distributions without motion correction, and the medians and interquartile ranges were 1273.5 [1248.3-1297.0] ms and 6.73 [6.1-7.9] ms, respectively.

^#^ Wilcoxon signed rank test was used in comparisons for native T1 and T1 fitting error; and paired t test for post-contrast T1 and ECV.

MoCo: motion correction to a MOLLI image series; Co-Reg: co-registration to a pair of native and post-contrast T1 maps. Fitting errors were calculated according to [16].

**Supplementary Table 2: Intra- and inter-observer reproducibility of ECV measurements without and with motion correction and co-registration.** Improved agreement was found using the proposed method as shown by the tighter 95% limits of agreement and higher intraclass correlation coefficients (ICC).

|  |  | **w/o MoCo** | | **w/ MoCo** | | **w/ MoCo & Co-Reg** | |
| --- | --- | --- | --- | --- | --- | --- | --- |
| **Intra-observer** | ECV [%] | Meas 1 | Meas 2 | Meas 1 | Meas 2 | Meas 1 | Meas 2 |
|  |  | 31.5 ± 3.6 | 32.0 ± 4.1 | 33.3 ± 7.6 | 33.7 ± 7.7 | 34.8 ± 7.1 | 34.8 ± 7.0 |
|  | 95% LOA | [-2.85 3.93] | | [-1.41 2.06] | | [-0.82 0.80] | |
|  | ICC | 0.904 [0.821 0.949] | | 0.993 [0.987 0.997] | | 0.998 [0.997 0.999] | |
| **Inter-observer** | ECV [%] | Obsr 1 | Obsr 2 | Obsr 1 | Obsr 2 | Obsr 1 | Obsr 2 |
|  |  | 31.5 ± 3.6 | 32.2 ± 4.2 | 33.3 ± 7.6 | 33.5 ± 7.1 | 34.8 ± 7.1 | 34.8 ± 7.0 |
|  | 95% LOA | [-3.40 4.72] | | [-1.48 1.85] | | [-1.11 1.06] | |
|  | ICC | 0.899 [0.803 0.927] | | 0.993 [0.987 0.997] | | 0.997 [0.994 0.998] | |
