# Supplementary figures and images for: Cardiac magnetic resonance T1 and extracellular volume mapping with motion correction and co-registration based on fast elastic image registration
Source: MAGMA. 2017 Dec 21;31(1):115–29. doi: 10.1007/s10334-017-0668-2 (PMC5813095; doi:10.1007/s10334-017-0668-2)

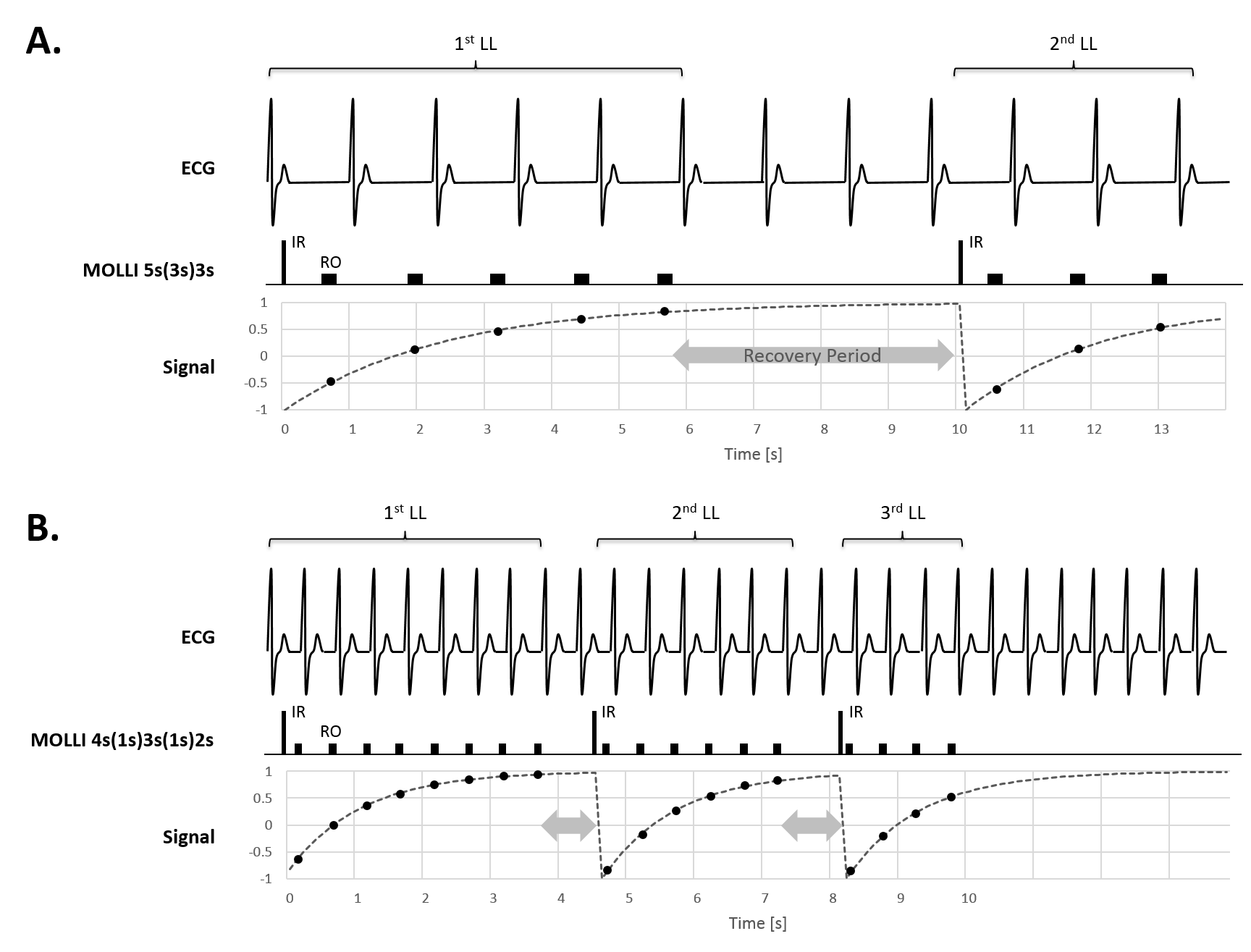

Supplement: Supplementary file 2 — Supplementary material 2 (PNG 115 kb) Supplementary Fig. 1 Schematic diagram of the modified look-locker inversion recovery (MOLLI) pulse sequence for myocardial T1 mapping. For native T1 mapping with a 5s(3s)3s scheme and enhanced or post-contrast mapping with a 4s(1s)3s(1s)2s scheme [13], different heartrates of 50 and 120 bpm were illustrated, respectively. The schemes including the look-locker (LL) periods and recovery intervals are essentially defined in terms of time periods with minimum durations in seconds. For instance, the number of single-shot image acquisitions during the LL periods is the ratio of defined minimum time duration to the R–R interval in seconds, rounded up to the next higher integer (e.g., 5 and 3 in A; 8, 6, and 4 in B), while recovery time is the minimum period between the end of the last acquired image to the next electrocardiogram (ECG)-triggered inversion recovery (IR) that encompasses a delay of ≥3 s for native (A) and of ≥1 s for postcontrast (B). Unlike the conventional beat-based scheme, the number of acquired images during each LL varies according to heartrate: less when R–R interval is longer; more when shorter. The exact scan time for breath-hold varies only slightly with the actual heartrate (13.2 s for 50 bpm using the native scheme and 10 s for 120 bpm using the enhanced scheme, as shown here), and is typically ~10 to 13 s. IR inversion recovery pulse, LL look-locker, RO signal readout [file 10334_2017_668_MOESM2_ESM.png]

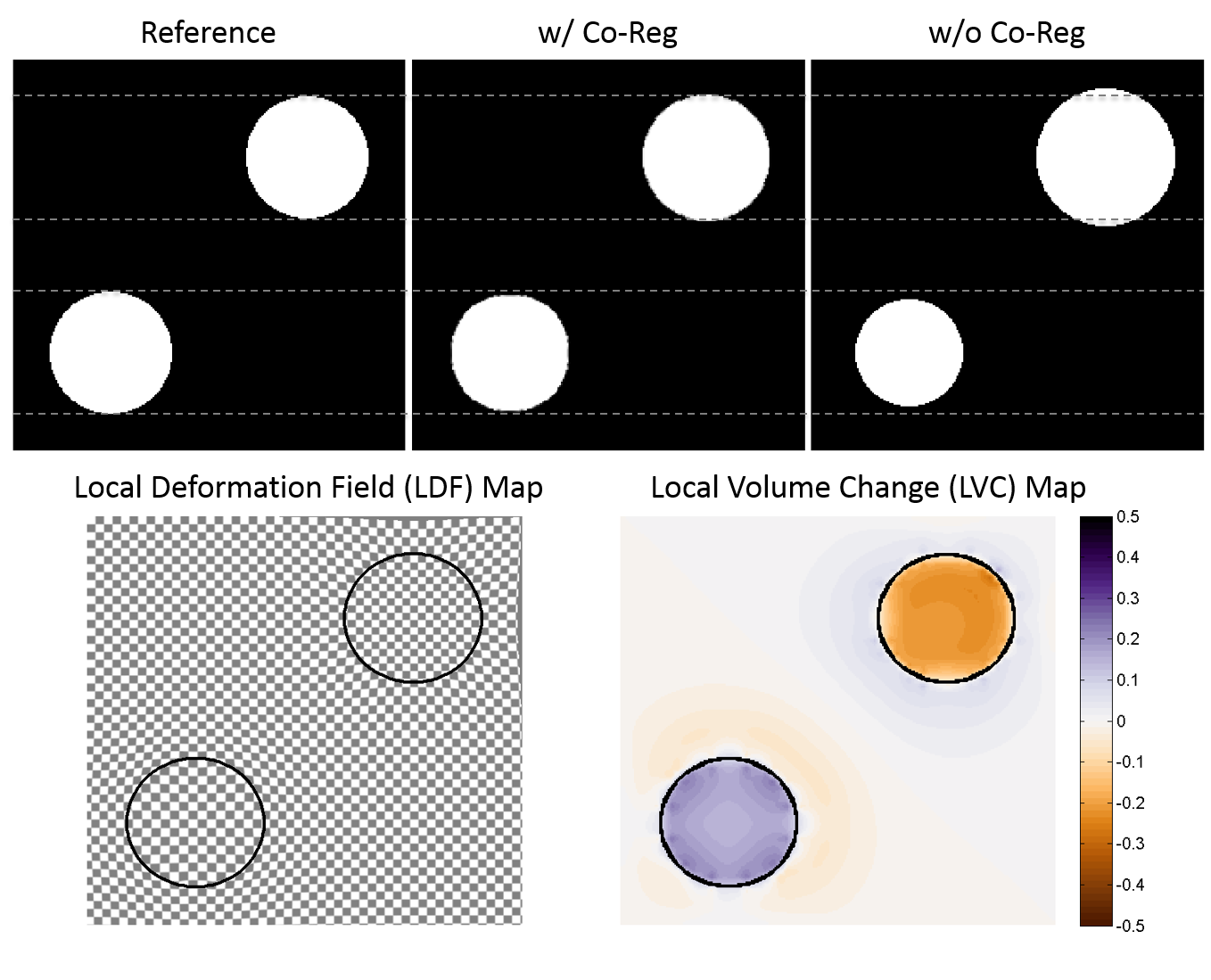

Supplement: Supplementary file 3 — Supplementary material 3 (TIFF 534 kb) Supplementary Fig. 2 Example of local deformation during image co-registration based on numeric simulation. A reference image (top left) was created with two circles of equal size. The image (top right) was created with larger and smaller diameters for the corresponding circles, respectively, and was registered to the reference image. The warped image after co-registration (top middle) showed compression and expansion from the corresponding circles, respectively. This can also be visualized by the locally deviated horizontal and vertical lines in the local deformation field (LDF) checkerboard map, as well as by the values in the local volume change (LVC) map, where a negative sign and brown color coding indicates compression (getting “hotter”), while a positive sign and blue color coding indicates expansion (getting “colder”). Such compression and expansion are purely image-based terms, which may be required for changes in the cross-sectional tissue area caused by motion. Both maps were overlaid with the iso-contours of the reference image. The same approach was applied to data and figures in this work [file 10334_2017_668_MOESM3_ESM.tif]
